# Emotional strategies to enhance resilience in patients with cancer: A scoping review

**DOI:** 10.1016/j.apjon.2025.100777

**Published:** 2025-08-22

**Authors:** Jiyin Zhang, Joyce Oi Kwan Chung, Sally Taylor, Janelle Yorke

**Affiliations:** a School of Nursing, The Hong Kong Polytechnic University, Hong Kong, China; b JBI Hong Kong Centre of Evidence-Based Healthcare Excellence, University of Adelaide, Australia; c Division of Nursing Midwifery and Social Work, School of Health Sciences, Faculty of Biology, Medicine & Health, The University of Manchester, Manchester, UK; d Christie Patient Centred Research (CPCR), The Christie NHS Foundation Trust, Manchester, UK

**Keywords:** Adults, Cancer, Emotional strategy, Mental health, Resilience, Scoping review

## Abstract

**Objective:**

To map and summarize existing evidence on emotional strategies recommended for enhancing resilience in cancer patients, identify research gaps and inform future research.

**Methods:**

Following Joanna Briggs Institute (JBI) guidelines, a comprehensive search was conducted across 11 databases in English and Chinese, supplemented by citation tracking and manual searches for published and unpublished studies. Studies focusing on adult cancer patients and describing emotional strategies to enhance resilience were included and critically appraised using tools appropriate to its design. Data including qualitative descriptions of emotional strategies, quantitative resilience-related emotional variables, and emotional intervention details were extracted and analysed with NVivo 15.

**Results:**

A total of 33 papers were included, primarily from China (*n* ​= ​16) and published as journal articles (*n* ​= ​30) with randomized controlled trial designs (*n* ​= ​14). Three key themes were identified: (a) emotion identification; (b) effective emotion regulation; and (c) emotional support from others. Emotional strategies were primarily implemented by nurses (*n* ​= ​11), delivered online (*n* ​= ​6) or face-to-face (*n* ​= ​13). “Positive” and “emotions” were the most frequently mentioned words.

**Conclusions:**

Emotion identification, effective emotion regulation, and emotional support from others are essential for enhancing resilience in cancer patients. Many promising strategies remain underutilized and require further validation.

**Systematic review registration:**

Open Science Framework (OSF) (DOI: 10.17605/OSF. IO/JBMZ9)

## Introduction

Cancer is a major public health, social, and economic challenge of the 21st century, with an estimated 19.96 million new cancer cases and nearly 9.74 million cancer-related deaths globally.^1^ Beyond the physical manifestations of cancer and its treatments, cancer patients commonly experience a broad range of psychological symptoms like anxiety, depression and concerns about recurrence, which substantially affect their quality of life and capacity to engage with medical care.^2^, ^3^, ^4^ Coping with these issues appropriately during survivorship can contribute significantly to the overall health and well-being of cancer patients.

Resilience is both a dynamic process and a psychosocial outcome characterized by healthy, adaptive functioning over time in the face of adversity. It encompasses an individual's capacity to bend without breaking.^5^ In the context of cancer, resilience reflects not only an endpoint but an ongoing recalibration in response to cancer-related events.^6^ It plays a crucial role for cancer patients, enabling them to cope with stress, actively engage in treatment, and achieve better clinical outcomes.^7^ According to the affect-regulation framework of psychological resilience, resilience has come from two major sources: the stress and coping approach and the emotion and emotion-regulation approach, guiding the development of the majority of resilience-enhancing interventions.^8^, ^9^, ^10^

The stress and coping approach focuses on managing externally valanced stress responses, emphasizes that the adaptive value of coping strategies depends on the context in which they are applied, requiring a fit between the characteristics of the stressor and the type of coping employed.^11^^,^^12^ In contrast, the emotion and emotion-regulation approach, as articulated in Gross's process model,^13^^,^^14^ is defined as “the process by which individuals influence which emotions they have, when they have them, and how they experience and express these emotions” . This approach focuses not on stress per se but on emotions elicited by both internal and external stimuli, whether positive or negative.^15^^,^^16^ For the purposes of this review, we define “emotional strategies” as the set of deliberate, emotion-focused strategies and resources that individuals employ or access to modulate the onset, intensity, duration, and expression of their emotions. These include antecedent-focused strategies such as emotion identification and understanding, response-focused strategies like expressive suppression, and external resources like seeking social or professional emotional support.^8^^,^^14^^,^^15^ It provides a more precise and emotion-cantered lens for developing interventions that support resilience and well-being in cancer patients.^17^

Importantly, emotional strategies are not only theoretically grounded but practically valuable. They can indirectly improve cognition and behaviour by enhancing emotional self-awareness and reducing distress, ultimately benefiting mental health.^15^^,^^18^, ^19^, ^20^ Nurses, who are often the most consistent point of contact for patients, play a critical role in delivering these strategies through education, communication, and psychosocial support.^21^ However, existing reviews have not explicitly summarized the specific roles nurses assume in implementing emotional strategies, nor have they addressed how nurses collaborate with other health care professionals in multidisciplinary interventions. Furthermore, cultural values, such as collectivism in East Asian societies, can shape whether patients prefer expressive or suppressive emotional strategies.^22^^,^^23^ These cultural dynamics may lead to variations in emotional strategies across different sociocultural contexts. Therefore, it is necessary to consider cultural factors within the scope of this review of emotional strategies to inform the future study. Despite the evident importance of emotional strategies in enhancing resilience, limited research has synthesized these strategies systematically, nor has it fully considered relevant implementation factors such as the roles of nurses or cultural contexts.

Currently, only two reviews have addressed resilience-enhancing interventions in cancer care, but both have notable limitations. For instance, a systematic review, meta-analysis, and meta-regression analyses synthesized randomized controlled trials (RCTs) on interventions to improve resilience.^24^ However, diversity in the content of the included intervention strategies led to high heterogeneity in this review, and excluded studies from Chinese databases, limiting its applicability to Chinese contexts.^24^ Another systematic review and network meta-analysis identified cognitive interventions, attention and interpretation therapy, and positive psychology as effective approaches for improving resilience but overlooked the utility of emotional interventions due to limited RCT availability.^25^ And both reviews excluded relevant studies that explored the development of emotion-related resilience strategies at a conceptual or methodological level using non-RCT research designs. In addition, both reviews failed to extract effective emotional strategy components from mixed interventions, contributing to high heterogeneity and limiting the identification of differential effects between stress-coping and emotion-focused strategies. To address these gaps, this study proposes a scoping review approach following the methods outlined by the Joanna Briggs Institute (JBI) Methods Manual for scoping reviews.^26^ By systematically mapping the evidence, this scoping review aims to provide a foundation for the development of targeted interventions that integrate effective emotional strategies to enhance resilience and improve mental health in cancer patients. The review question is: What emotional strategies are available to enhance resilience in adult cancer patients?

## Methods

The scoping review was conducted following the JBI methodology for scoping reviews.^26^ The study was registered before data analysis with Open Science Framework (OSF) (DOI: 10.17605/OSF.IO/JBMZ9).

### Eligibility criteria

Our eligibility criteria were conceptualized using the participants, concept and context, as follows: (1) participants: adult cancer patients (age ≥ 18 years old). Patients with severe mental disorders currently receiving psychiatric treatment were excluded; (2) concept: literature should focus on emotional strategies aimed at enhancing individual psychological resilience. Emotional strategies should view emotions as responses to stimuli, involving approaches which specifically focus on emotions; (3) context: any health care setting and any study design were eligible. We included all English- and Chinese-language publications from database inception through November 8, 2024, and performed citation searches of related reviews.

### Search strategy

The search strategy aimed to locate both published and unpublished studies. The search strategy was first developed by our researchers, then a librarian was consulted to refine the search strategy (Appendix A). Published papers were searched through 11 electronic databases (7 English databases and 4 Chinese Databases) - PubMed, Embase, American Psychological Association PsycInfo (via ProQuest), Web of Science, Cochrane Library, CINAHL, Scopus, Wanfang Database, CNKI (China National Knowledge Infrastructure), SinoMed (Chinese Biomedical Literature Service System) and VIPC. A strategy for retrieving unpublished papers involves searching specialized databases, ProQuest Dissertations & Theses, using a search engine, Google Scholar, with tailored keywords. Exploring books and manuals using tailored keywords via The Hong Kong Polytechnic University Library. The reference list of all relevant systematic reviews, meta-analysis and traditional reviews were screened for additional studies.

### Data screening and selection

Two reviewers applied the eligibility criteria to identify the relevant studies. Following the search, all identified citations were collated and uploaded into EndNote 21 and duplicates were removed. Following a pilot test, titles and abstracts were screened by two independent reviewers for assessment against the inclusion criteria for the review. At the same time, a manual search for duplicates was conducted and duplicates were removed. The full text of selected citations was assessed in detail against the eligibility criteria by two independent reviewers. Reasons for the exclusion of sources of evidence in full text that do not meet the eligibility criteria were recorded and reported in the scoping review. Any disagreements that arose between the reviewers at each stage of the selection process were resolved through discussion. The results of the search and the study inclusion process were reported in full in the final scoping review and presented in a Preferred Reporting Items for Systematic Reviews and Meta-Analyses (PRISMA) 2020 flow diagram for new systematic reviews which included searches of databases, registers and other sources.^27^

### Data extraction

Data were extracted from papers included in the scoping review by two independent reviewers using NVivo 15. Specific data extraction included: (a) Author(s), year of publication, and country; (b) aims of the study; (c) article type; (d) methodology (study design, setting and participants); and (e) key findings relevant to the scoping review questions (Table B.1 in Appendix B). For data extraction of emotional strategies, we extracted descriptions of strategies aimed at improving resilience from qualitative studies. For quantitative studies, we extracted descriptions of variables positively associated with resilience and emotional strategies that contributed to the overall effect on resilience in the trials.

### Data analysis and synthesis

The main characteristics and the key findings of the included documents are presented in Table B.1 in Appendix B. Descriptive qualitative content analysis was performed to obtain in-depth analyses.^26^ Data analysis for the emotional strategies to enhance resilience in cancer patients was conducted using qualitative content analysis aimed at documenting the main themes of the descriptions in question. Data on emotional strategies identified were imported into NVivo 15 qualitative software for analysis. The texts used to conduct the content analysis of the emotional strategies are presented in Table C in Appendix C. According to the context or explanation provided by the document authors, contents that expressed similar meanings were labelled a code. After that, categories were created by identifying links between these codes and sorting codes that expressed similar meanings. Finally, categories were refined into a set of overriding themes. We evaluated the quality based on the type of documents included. The bias of RCTs or protocol was evaluated using Cochrane Risk of Bias 2 (RoB 2.0).^28^ Analytical cross-sectional studies were assessed using the JBI critical appraisal checklist for analytical cross-sectional studies.^29^ Qualitative studies were assessed using the JBI critical appraisal checklist for qualitative research,^30^ and mixed methods study was assessed using Mixed Methods Appraisal Tool (MMAT), version 2018.^31^ We did not evaluate the book included owing to the lack of appropriate tools for its critical appraisal.

## Results

### Literature search

**Fig. 1:**
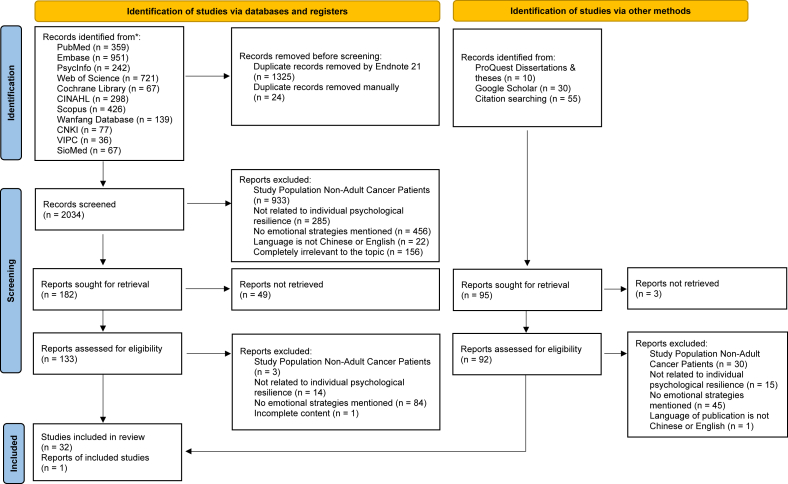
PRISMA flow diagram of the selection of sources of evidence.

A total of 3383 records from 11 databases and 95 records from other sources were retrieved according to the search strategy. After removing 1349 duplicate documents, 2034 titles and abstracts were screened, and 225 full texts were assessed, a total of 33 fulfilled our eligibility criteria (Fig. 1).

### Descriptive characteristics of the documents

**Fig. 2:**
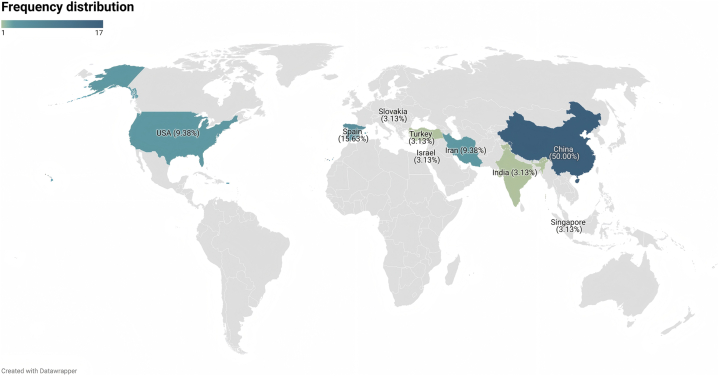
Choropleth of document distribution by geographic region.

The included documents were derived from nine different countries, with the majority being from China (*n* ​= ​17), followed by Spain (*n* ​= ​5), Iran (*n* ​= ​3), USA (*n* ​= ​3), India (*n* ​= ​1), Israel (*n* ​= ​1), Singapore (*n* ​= ​1), Slovakia (*n* ​= ​1), and Turkey (*n* ​= ​1) (Fig. 2). There were 31 journal articles, one conference paper and one book chapter. In terms of study design, 15 RCTs, 10 quantitative studies, five qualitative studies, one mixed-method study, one RCT protocol, and one book chapter were included. Twenty-two studies were conducted in a hospital setting, three were conducted in a cancer or care association and three were conducted in a cancer center setting. Two studies were conducted across multiple settings and three did not focus on specific settings. Most studies focused on breast cancer (*n* ​= ​12), followed by the mixed cancer types (*n* ​= ​8), colon/colorectal cancer (*n* ​= ​3), liver cancer (*n* ​= ​3), gastric cancer (*n* ​= ​1), esophageal cancer (*n* ​= ​1), gynecological cancer (*n* ​= ​1), lung cancer (*n* ​= ​1), nasopharyngeal cancer (*n* ​= ​1), ovarian cancer (*n* ​= ​1) prostate cancer (*n* ​= ​1) and acute leukemia (*n* ​= ​1). Two studies specifically targeted young adults as the study population. Details of the included documents are provided inTable B.1 in Appendix B.

### Quality assessment

The quality score of 10 analytical cross-sectional studies ranged from 3/8 to 8/8 (Table B.2 in Appendix B). The most common source of methodological limitation was the lack of clarity or absence of strategies to deal with confounding factors, with four studies rated as “unclear” and two rated as “no” on this item, which substantially impacted overall quality ratings. The quality score of five qualitative studies ranged from 3/10-10/10. The main weakness affecting study quality was the lack of consideration of the researcher's influence on the research and vice versa, with this criterion rated “no” in three out of five studies. The only mixed methods study included received a score of 4/5 (Table B.3 in Appendix B), with the main shortcoming being a lack of discussion on divergences or inconsistencies between qualitative and quantitative findings. Among the 14 randomized controlled trials and one protocol assessed using the Cochrane Risk of Bias tool, one study was rated as having “low risk” and three as “high risk of bias.” The most frequently observed high-risk domain was the randomization process. These results are summarized in Fig. B1, which presents the overall risk of bias across studies, and detailed ratings for each domain of the 14 randomized controlled trials are shown in Fig. B2 in Appendix B as only one protocol was included, its domain-level ratings are not displayed separately in Fig. B2.

### Thematic findings of emotional strategies to enhance resilience in cancer patients

**Fig. 3:**
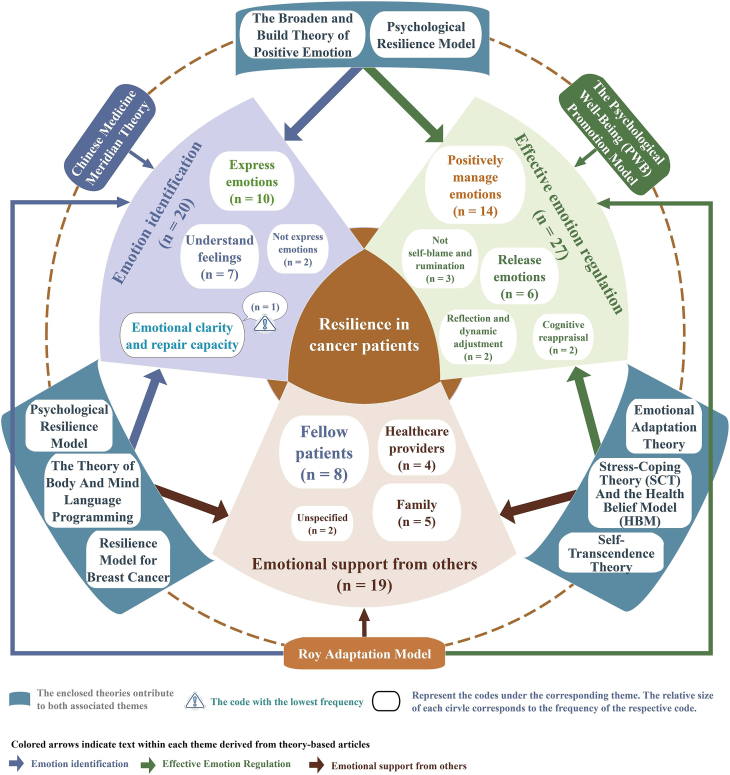
Thematic map of emotional strategies to enhance resilience in cancer patients.

**Fig. 4:**
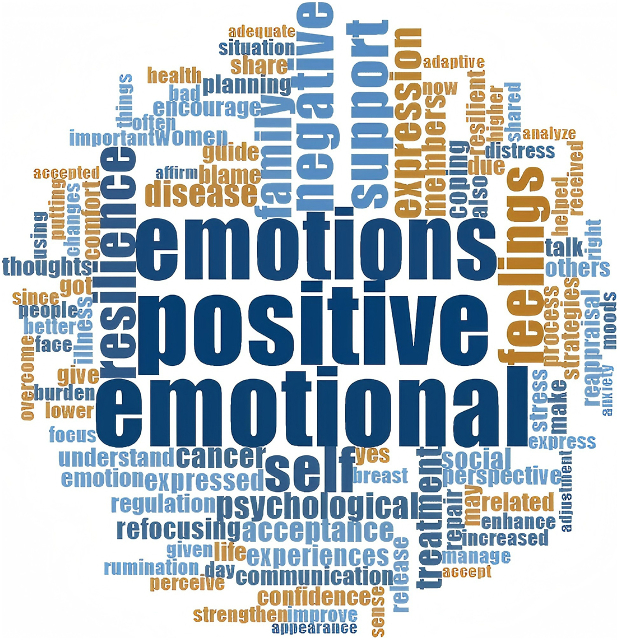
Word cloud of emotional strategies in included documents.

In this review, relevant textual descriptions of emotional strategies within the included documents were analysed through inductive node coding: for example, the prompt “the patient is instructed to self-ask the following questions: Do I have negative feelings right now? If yes, what are they? Do I have positive feelings right now? If yes, what are they?”^32^ was coded as “understand feelings,” “promoting change towards positive attitudes, introducing emotional communication skills and assertiveness, adequate expression and repair of negative emotions …”^33^ was coded as “positively manage emotions,” and “family support is a crucial factor in coping with breast cancer, providing emotional comfort and reassurance.”^34^ was coded as “emotional support from family.” A total of 66 nodes were generated. By comparing node meanings, those reflecting “recognition, perception, judgment, and attribution of one's emotions, either independently or under guidance” were clustered as emotion identification; nodes denoting “modulation of emotional intensity or duration through cognitive, behavioural, or environmental adjustments, independently or with guidance” were grouped as effective emotion regulation; and those capturing “fulfilment of emotional needs through verbal, behavioural, or resource-based support in interpersonal interactions” were classified as emotional support from others. Accordingly, these 66 nodes were organized into three overarching themes: (1) emotion identification; (2) effective emotion regulation; and (3) emotional support from others, and visualized in a thematic concept map (Fig. 3). Eleven documents mentioned the implementer of the strategy as a nurse, Seven as a psychologist and three as an internist. The delivery modes of intervention were mentioned 14 times for online and seven times for face-to-face. Note that a single article containing information related to multiple themes may have been grouped into more than one theme. The content of the original text used to do the thematic analysis of the emotional strategies is provided in Appendix C. Fig. 4 presents the word cloud generated from the text used for emotional strategies thematic analysis. “Positive” and “emotional” were the most frequently mentioned words.

### Emotional strategies

#### Emotion identification

A total of 18 documents with 20 nodes referred to emotional identification as a means to enhance resilience in cancer patients. Under this theme, one study referring to emotional clarity and repair capacity, demonstrates that breast cancer patients who perceive their emotions clearly and trust in their abilities to repair their emotional states are more strengthened and resilient.^35^ Ten documents mentioned that expressing emotions whether using verbal or non-verbal forms can enhance resilience in cancer patients.^33^^,^^36^, ^37^, ^38^, ^39^, ^40^, ^41^, ^42^, ^43^, ^44^ However, two documents made the opposite point that not expressing emotions is more likely to improve resilience in cancer patients.^45^^,^^46^ Seven documents mentioned understanding feelings, including awareness of emotions and the specific feelings.^32^^,^^36^^,^^45^^,^^47^, ^48^, ^49^, ^50^

#### Effective emotion regulation

A total of 22 documents with 27 nodes mentioned that effective emotion regulation can improve resilience in cancer patients. Under this theme, the classifications include: positive management of emotions (*n* ​= ​14),^7^^,^^33^^,^^34^^,^^39^^,^^40^^,^^46^^,^^51^, ^52^, ^53^, ^54^, ^55^, ^56^, ^57^, ^58^ cognitive reappraisal (*n* ​= ​2),^41^^,^^59^ not self-blame and rumination (*n* ​= ​3)^7^^,^^53^^,^^54^ and reflection and dynamic adjustment (*n* ​= ​2).^32^^,^^43^ Positive management of emotions was the most mentioned node which refers to addressing emotions in a positive way including acceptance, positive refocusing, and positive processing of emotions. Cognitive reappraisal emphasis focuses on generating benign or positive interpretations or perspectives on stressful situations and is an effective way for resilient patients to deal with negative emotions.^41^^,^^59^ Self-blame and rumination are seen as approaches used by people with low emotion regulation ability. It makes it difficult for patients to regulate their emotions under stress and is associated with low resilience.^7^^,^^53^^,^^54^ The theme of reflection and dynamic adjustment views resilience as a dynamic process and aims to guide patients to reflect on past successful emotion regulation experiences to further improve their resilience.^32^^,^^43^ A total of six documents referred to release emotions as a means to enhance resilience in cancer patients.^34^^,^^42^^,^^46^^,^^60^, ^61^, ^62^ It emphasizes that cancer patients should allow their emotions to surface spontaneously and know how to release them. The ways in which emotions were released varied across documents, including confiding in others or participating in other activities for example.

#### Emotional support from others

Emotional support was mentioned in 19 nodes in 14 documents as an effective way to improve resilience.^32^^,^^34^^,^^37^^,^^38^^,^^40^^,^^44^^,^^46^^,^^48^^,^^49^^,^^52^^,^^57^^,^^61^, ^62^, ^63^ Emotional support can come from fellow patients (*n* ​= ​8); ^32^^,^^40^^,^^46^^,^^48^^,^^49^^,^^57^^,^^61^^,^^62^ family (*n* ​= ​5);^34^^,^^44^^,^^46^^^,^^^48^^,^^61^ health care providers (*n* ​= ​4);^34^^,^^37^^,^^46^^,^^61^ two studies mentioned emotional support but did not specify its source.^38^^,^^52^ Emotional support can be provided in a variety of ways, including group experiences, health coaching, emotional sharing sessions, etc.

### Implementer of emotional strategies

Not all documents mentioned who would implement emotional strategies. In extracting this theme, for the randomized controlled trials we extracted the role of implementing the intervention, and in the non-intervention trial documents we extracted the implementers recommended by the authors. Nurses were the most frequently mentioned implementers and were involved in the development and implementation of the intervention program, as well as its evaluation and monitoring.^32^^,^^37^^,^^42^, ^43^, ^44^^,^^46^^,^^48^, ^49^, ^50^^,^^52^^,^^61^ Psychologists were also frequently noted as implementers, and in the seven documents where psychologists were mentioned as implementers of emotional strategies, all involved the need for professional psychotherapy (e.g., relaxation therapy, psychological counselling, etc.).^33^^,^^39^^,^^43^^,^^49^^,^^52^^,^^58^^,^^61^ In addition, three documents mentioned the involvement of internists in the implementation of emotional strategies.^39^^,^^52^^,^^61^

### Modes of delivering emotional strategies

The modes of delivery emotional strategies were identified as online and face-to-face. Seven documents referred to online modes, which included education on emotion regulation strategies, recording of emotions, telephone and video follow-up, etc.^32^^,^^39^^,^^48^^,^^49^^,^^55^^,^^57^^,^^61^ Fourteen documents referred to face-to-face modes, where interventions such as emotional sharing sessions and counselling could not be carried out online.^32^^,^^36^^,^^37^^,^^39^^,^^43^^,^^44^^,^^48^^,^^50^^,^^52^^,^^61^^,^^62^

### Theoretical frameworks guiding the study

A total of 11 distinct theoretical frameworks guided the included studies. Eight were specifically applied to developmental intervention programs: (1) The Psychological Well-Being (PWB) Promotion Model;^55^ (2) Chinese Medicine Meridian Theory;^50^ (3) Emotional Adaptation Theory;^62^ (4) Positive Psychology;^33^ (5) Resilience Model for Breast Cancer (RM-BC);^49^ (6) Roy Adaptation Model (RAM);^32^ (7) Self-Transcendence Theory^52^ and (8) The Theory of Body And Mind Language Programming.^44^ In included qualitative studies, Psychological Resilience Model^38^ informed both research design and data analysis, while Stress-Coping Theory (SCT) And the Health Belief Model (HBM)^34^ structured the semi-structured interview protocols. The included cross-sectional study employed the Broaden and Build Theory of Positive Emotion,^41^ hypothesizing that patients with higher resilience would be more likely to express positive emotions, engage in positive reappraisal, and cultivate a greater sense of inner peace and meaning. The connection of these theories to the themes of emotional strategies is shown in Fig. 3.

## Discussion

### Main findings

This study synthesized 33 studies to summarize and map the recommended emotional strategies to improve resilience in adult cancer patients. All these documents highlighted the potential and identified value of emotional strategies to enhance resilience in cancer patients. Three themes were identified, including emotion identification; effective emotion regulation; and emotional support from others. All of them are important in enhancing resilience of cancer patients.

Emotion identification refers to the accurate identification and labelling of emotions, and involves awareness of emotional experiences and the ability to label these emotions appropriately.^64^ We have grouped four categories under this theme. First, emotional clarity and repair capacity, despite being mentioned only once, direct effects of this study's result showed that greater resilience is predicted through the increases in the levels of both emotional clarity and mood repair.^35^ However, no clinical trials have examined the effect of helping cancer patients clearly perceive their emotions and strengthen their ability to regulate emotional states and resilience, which may require further validation in future studies. It is interesting that two conflicting strategies of “express emotions” and “not express emotions” are identified in the theme of emotion identification. Although most studies have concluded that expressing emotions is an effective strategy for enhancing resilience, there are still two articles that have concluded that not expressing emotions is an effective strategy.^45^^,^^46^ One study mentioned that “social context surrounding emotion expression plays an important role in facilitating adjustment for younger breast cancer patients,” and noted that the participants in this cross-sectional study were recruited via social media outlets requiring them to “like” or “follow” specific cancer-related organizations.^45^ This recruitment method may have selectively attracted individuals who seek emotional support online rather than through face-to-face interactions, suggesting that recruitment mode and engagement with emotional content can systematically influence findings. Nevertheless, it also reminds us that younger cancer patients may avoid expressing emotions in person to prevent burdening family and friends, underscoring how perceived social support shapes the appropriateness of different emotional strategies.^65^ In the other included qualitative study, where “not expressing emotions” was also coded as an effective emotional strategy for enhancing resilience, participants explained that they “do not want the expression of emotions negatively affecting family and friends,” which may be attributable to different social context.^46^ A meta-analysis supports that expressive suppression may have a detrimental effect on resilience in individuals with western cultural values but no effect on resilience in individuals with eastern cultural values.^66^ Furthermore, cultural norms around emotional display influence whether expression or suppression is adaptive. Research in collectivist cultures, which prioritize group harmony, shows that expressive suppression is less harmful or even beneficial in some Asian samples.^67^ However, few studies have incorporated these cultural considerations into their intervention designs. Altogether, the conflicting findings on “express emotions” versus “not express emotions” highlight the nuanced role of patient demographic background, social context and recruitment mode in shaping emotional strategies for resilience. The included studies that promotes emotion expression provides some solutions to minimize social impact, like a more private written emotion diary to help express emotions^36^^,^^38^^,^^43^ and counselling.^39^^,^^40^ Future studies may benefit from considering the social context when developing emotional strategies for cancer patients. Understanding Feelings was the second most frequently mentioned category in this theme (following expressing emotions) and was seen as perceiving and understanding the emotions present in oneself, whether negative or not. Understanding Feelings can help patients to better adopt effective, targeted coping measures also facilitates patients to be more able to reduce the impact of cognitive biases typical of chronic disease management and identify and focus on supportive resilience resources,^17^ thus increasing resilience. Therefore, a better understanding of feelings can help cancer patients to better classify and express their emotions, thus improving their resilience. This comes from the emotional complexity of patients, also known as granularity,^64^ which could be one of the targets for future interventions to improve patient resilience.

In addition to emotion identification, engaging in effective emotion regulation is also a meaningful emotional strategy that is effective in increasing resilience in cancer patients. These included positive management of emotions (*n* ​= ​13), cognitive reappraisal (*n* ​= ​2), not self-blame and rumination (*n* ​= ​3), reflection and dynamic adjustment (*n* ​= ​2) and release emotions (*n* ​= ​6). Positive management of emotions is the most frequently mentioned of all emotional strategies. “Positive” is also the most frequent word in the word cloud. It is based on the theories of positive psychology.^68^ Individuals who can positively regulate their emotions pay attention to emotion-provoking situations, using relevant resources and coping measures appropriately.^69^ This can better help cancer patients build resilience. It has often been used as an important intervention unit in many studies, teaching patients positive emotional coping approaches and strategies, all of which have been shown to meaningfully enhance the resilience of cancer patients.^33^^,^^70^ “Cognitive reappraisal” and “not self-blame and rumination” were derived from cross-sectional studies discussing the correlation between emotion regulation skills and resilience. They included as emotional strategies in this scoping review for the reason that in the literature included, the reappraisal is specific to the emotion rather than the event itself, highlighting that emotion regulation with higher scores in cognitive reappraisal and lower scores in self-blame and rumination, is highly correlated with higher levels of resilience.^7^^,^^41^^,^^53^^,^^54^^,^^59^ In addition, reflection and dynamic adjustment deserve to be noticed in the improvement of resilience, which sees resilience as a dynamic process, reviewing previous experiences of managing emotions can effectively help cancer patients enrich their coping strategies and regain a sense of internal control and strength, thereby enhancing resilience.^71^ Despite strong evidence correlating emotion regulation skills with resilience, few interventional studies directly address strategies like cognitive reappraisal or dynamic emotional adjustment. Rigorous randomized controlled trials are needed to validate and refine these strategies for practical application. Moreover, emotional release has been mentioned in many studies as an effective way to increase resilience in cancer patients. However, most of the literature does not specify what modalities were used to help patients release their emotions. In the descriptions in some included documents, emotional release is described as “personalized” and “varied”.^42^^,^^46^^,^^61^^,^^62^ It's worth noting that some methods involving professional psychological techniques, such as relaxation therapy and emotional catharsis, were recommended to be carried out by a professional practitioner with emotional support from others.^61^

Social support, as an important source of resilience, plays an important role in increasing levels of resilience and quality of life in cancer patients.^72^^,^^73^ Positive emotional support helps patients be resilient. The main sources of emotional support mentioned in the included documents were medical support from professionals, allowing patients to construct confidence in the treatment of their disease; sharing from other patients, allowing them to develop stronger emotional empathy; and support and companionship from the patient's family and friends, allowing patients to cope more effectively with the stresses that come from cancer and treatment. Although we undertook rigorous procedures to maintain thematic clarity, we acknowledge that some original descriptions are vague and lack explicit operational guidelines, which may have led to overlap during thematic extraction. For example, “record the day's emotions and behaviors on a daily basis,”^36^ offers no indication of whether patients are documenting emotion identification or enacting behavioural regulation strategies, nor does it include exemplar diary entries. After thorough discussion among multiple researchers, and given the subsequent passage that “health care personnel, through debate and in-depth communication, enable patients to perceive their own irrational emotions and establish new rational beliefs,”^36^ we ultimately coded this excerpt under emotion identification. We therefore concede that incomplete reporting in the source material may have introduced ambiguity and overlap between our defined themes. Future research should report clear and detailed operational protocols to minimize ambiguity and thereby enhance the replicability and scalability of emotional interventions.

The modes of delivery emotional strategies were identified as online and face-to-face. Both approaches are good for enhancing resilience but are not limited to one mode of delivery in an intervention program. For example, using a more convenient and accessible online mode to enhance awareness of one's own emotions, facilitating the expression of emotions, teaching emotion regulation strategies, using face-to-face sessions for group sharing sessions to provide further emotional support. For the implementers of the intervention, we found the largest number of studies that included nurses as implementers of the strategy in the included documents. Nurse plays an important role in the symptom management for cancer patients, suggesting the potential of nurses' role in emotional support.^74^^,^^75^ However, where specialized psychotherapeutic techniques were present in the intervention program, the document advocated that the intervention be delivered by a psychologist. In addition, physicians also play an important role in implementation. It indicates that a multi-disciplinary approach could be beneficial, and nurses can be the main implementers of intervention programs to enhance the resilience of cancer patients. Additionally, eleven theoretical frameworks were used to guide the included studies. We found that only two theoretical frameworks were directly related to emotions, namely, emotion adaption theory^62^ and the Broden and build theory of positive emotion.^41^ These frameworks offer valuable insights into how positive emotions and emotional adaptation foster resilience. Future research should explore integrating emotion-focused frameworks into intervention design, potentially offering a robust theoretical basis for enhancing resilience in cancer patients.

While the included studies offered important insights, the overall quality of evidence varied across study designs. Several analytical cross-sectional studies lacked clearly stated strategies to address potential confounding factors, which may weaken the validity of their findings. In the qualitative studies, limited attention was given to researcher reflexivity, a critical aspect that may influence data collection and interpretation. The only mixed methods study did not sufficiently address inconsistencies between its qualitative and quantitative components. Among the randomized controlled trials, although most were of moderate to high quality, the randomization process was frequently rated as high risk or unclear. These methodological weaknesses highlight the need for more rigorous study design and transparent reporting in future research to enhance the reliability and applicability of findings on emotional strategies that promote resilience in cancer patients.

### Implications for nursing practice and research

The findings offer a clear framework for structuring emotional strategies to enhance resilience in cancer patients across the three core domains: emotion identification, effective emotion regulation and emotional support from others. Nurses should be encouraged to assess patients’ emotional awareness and regulation abilities as part of routine care and provide individualized emotional support accordingly. The manuscript further emphasizes the need to consider social context in emotional strategies, as the adoption of these strategies, whether expressing or suppressing emotions, often depends on relational and cultural factors and patient demographic background. In clinical practice, facilitating culturally sensitive communication about emotions and supporting patients in choosing appropriate emotion expression methods are recommended. A key gap identified is the lack of intervention studies that leverage patients' strengths in recognizing and categorizing their emotions to enhance resilience through the adoption of effective emotional strategies. Additionally, there is a lack of high-quality evidence validating the effectiveness of some emotional strategies. Future research should focus on developing context-sensitive approaches that integrate emotional identification, regulation, and support while rigorously testing their effectiveness. Moreover, incorporating technology-assisted delivery methods, such as online platforms, could further improve resilience and psychological well-being in cancer patients. Interprofessional collaboration, particularly involving nurses, psychologists, and physicians, is essential for the successful implementation of such interventions.

### Limitations

Our scoping review process had several limitations. While we conducted a quality evaluation of the included literature, which is relatively uncommon in scoping reviews,^76^ we were unable to assess the quality of the included books due to the limitations of existing tools and methods. We did not exclude studies based on the results of the quality evaluation, as we aimed to provide a comprehensive overview of the knowledge in this field. By design, the scoping review was unable to compare the specific effects of various emotional strategies on resilience in cancer patients, which represents a methodological limitation. Furthermore, the reporting of emotional strategies in the literature was inconsistent, limiting our ability to analyse these strategies in depth. Lastly, to ensure the integrated emotional strategies were fully applicable to adult cancer patients, we excluded studies involving mixed populations, such as those including both adolescents and young adults, or studies focusing on patients paired with their primary caregivers. Future research could explore emotional strategies tailored to cancer patients across different age groups to provide more precise and targeted intervention recommendations.

## Conclusions

This scoping review provides a comprehensive analysis of emotional strategies to enhance resilience for cancer patients, synthesizing findings from 33 documents across nine countries. The studies demonstrate diverse methodologies and settings, highlighting the significant role of emotion-focused interventions in improving patient resilience. Three themes were demonstrated in the review including emotion identification, effective emotion regulation and emotional support from others. Future research and practice should focus on developing personalized and multidisciplinary emotion-focused interventions that leverage patients' strengths in recognizing and categorizing their emotions to enhance resilience. Given that some promising strategies (e.g., emotional clarity) were primarily derived from correlational and qualitative studies, rigorous intervention research is needed to validate their effectiveness. Additionally, future studies should address gaps in specific cancer populations, such as young adults, and further explore the mechanisms underlying effective emotional strategies.

## CRediT authorship contribution statement

**Jiyin Zhang:** Conceptualization, Methodology, Literature Search, Data Extraction, Formal Analysis, Writing – Original Draft. **Joyce Oi Kwan Chung:** Conceptualization, Methodology, Literature Search, Data Extraction, Formal Analysis, Supervision, Writing – Review & Editing. **Sally Taylor:** Conceptualization, Writing – Review & Editing. **Janelle Yorke:** Conceptualization, Supervision, Writing – Review & Editing. All authors have read and approved the final manuscript.

## Ethics statement

Not required.

## Data availability

Data availability is not applicable to this article as no new data were created or analyzed in this study.

## Declaration of generative AI and AI-assisted technologies in the writing process

During the preparation of this work, the authors used ChatGPT only for grammar and language refinement. No content, data analysis, or interpretation was generated by AI. After using this tool, the authors reviewed and edited the content as needed and take full responsibility for the content of the publication.

## Funding

This study received no external funding.

## Declaration of competing interest

The authors declare no conflict of interest.
