# Supplementary material for: Emotional strategies to enhance resilience in patients with cancer: A scoping review
Source: Asia Pac J Oncol Nurs. 2025 Aug 22;12:100777. doi: 10.1016/j.apjon.2025.100777 (PMC12802108; doi:10.1016/j.apjon.2025.100777)
Supplement: Multimedia component 1 [file mmc1.docx]

**Appendix A: Search Strategy**

Search database: PubMed, Embase, American Psychological Association PsycInfo (via ProQuest), Web of Science (1966 to present), Cochrane Library, CINAHL, Scopus, Wanfang Database, CNKI (China National Knowledge Infrastructure), VIPC. SinoMed.

Search conducted from October to 8 November 2024

**Database:**

1. PubMed

| Search ID# | Search Terms | Results |
| --- | --- | --- |
| #1 | "Neoplasms"[Mesh] OR "Oncology" OR "Tumor" OR "Malignancy" OR "Cancer" | [5,831,808](https://pubmed.ncbi.nlm.nih.gov/?term=%22Neoplasms%22%5BMeSH%5D+OR+%22Oncology%22+OR+%22Tumor%22+OR+%22Malignancy%22+OR+%22Cancer%22&sort=relevance) |
| #2 | “Resilience, Psychological” [Mesh] OR “Emotional Resilience” | [10,606](https://pubmed.ncbi.nlm.nih.gov/?term=%E2%80%9CResilience%2C+Psychological%E2%80%9D+%5BMeSH%5D++OR+%E2%80%9Cemotional+resilience%E2%80%9D&sort=relevance) |
| #3 | "Emotional Strategy" OR "Emotion Regulation" OR "Emotional Regulation" OR "Emotional Therapy" OR "Mindfulness" OR "Emotion Management" OR "Emotional Coping" OR "Affective Regulation" OR "Affective Strategy" OR "Psychological Adaptation" OR "Stress Management" OR "Emotional Awareness" OR "Emotional Processing" OR "Adaptive Skills" OR "Emotional Training" OR "Emotion Labelling" OR "Emotional Differentiation" | 151,386 |
| #4 | "Emotions" [Mesh] OR Emotion* OR Psycho* OR Affect* | 5,074,149 |
| #5 | Strateg* OR Tactic* | 1,787,314 |
| #6 | #1 AND #2 AND #3 | 304 |
| #7 | #1 AND #2 AND #4 AND #5 | 131 |
| #8 | #6 OR #7 | 359 |

1. Embase

| Search ID# | Search Terms | Results |
| --- | --- | --- |
| #1 | 'Neoplasm'/Exp OR 'Oncology' OR 'Tumor' OR 'Malignancy' OR 'Cancer' | [8,571,616](https://www-embase-com.ezproxy.lb.polyu.edu.hk/) |
| #2 | 'Psychological Resilience'/Exp OR 'Emotional Resilience' | 74,485 |
| #3 | “Emotional Strategy” OR “Emotion Regulation” OR “Emotional Regulation” OR “Emotional Therapy” OR “Mindfulness” OR “Emotion Management” OR “Emotional Coping” OR “Affective Regulation” OR “Affective Strategy” OR “Psychological Adaptation” OR “Stress Management” OR “Emotional Awareness” OR “Emotional Processing” OR “Adaptive Skills” OR “Emotional Training” OR “Emotion Labelling” OR “Emotional Differentiation” | [68,891](https://www-embase-com.ezproxy.lb.polyu.edu.hk/) |
| #4 | 'Emotion'/Exp OR Emotion* OR Psycho* OR Affect* | 6,214,172 |
| #5 | Strateg* OR Tactic* | 2,203,313 |
| #6 | #1 AND #2 AND #3 | 347 |
| #7 | #1 AND #2 AND #4 AND #5 | 683 |
| #8 | #6 OR #7 | 951 |

1. PsycINFO (via ProQuest)

| Search ID# | Search Terms | Results |
| --- | --- | --- |
| S1 | MAINSUBJECT.EXACT("Neoplasms") OR Oncology OR Tumor OR Malignancy OR Cancer | 127,480 |
| S 2 | MAINSUBJECT.EXACT("Resilience (Psychological)") OR (Emotional Resilience) OR (Resilience) | 50,142 |
| S3 | “Emotional Strategy” OR “Emotion Regulation” OR “Emotional Regulation” OR “Emotional Therapy” OR “Mindfulness” OR “Emotion Management” OR “Emotional Coping” OR “Affective Regulation” OR “Affective Strategy” OR “Psychological Adaptation” OR “Stress Management” OR “Emotional Awareness” OR “Emotional Processing” OR “Adaptive Skills” OR “Emotional Training” OR “Emotion Labelling” OR “Emotional Differentiation” | 72,273 |
| S4 | Emotion* OR Psycho* OR Affect* | 3,176,763 |
| S5 | Strateg* OR Tactic* | 473,702 |
| S6 | S1 AND S2 AND S3 | 109 |
| S7 | S1 AND S2 AND S4 AND S5 | 157 |
| S8 | S6 OR S7 | 242 |

1. Cochrane Library

| Search ID# | Search Terms | Results |
| --- | --- | --- |
| #1 | 'Neoplasm'/Exp OR 'Oncology' OR 'Tumor' OR 'Malignancy' OR 'Cancer' | [127536](https://www-embase-com.ezproxy.lb.polyu.edu.hk/) |
| #2 | 'Psychological Resilience'/Exp OR 'Emotional Resilience' | [4308](https://www-embase-com.ezproxy.lb.polyu.edu.hk/) |
| #3 | “Emotional Strategy” OR “Emotion Regulation” OR “Emotional Regulation” OR “Emotional Therapy” OR “Mindfulness” OR “Emotion Management” OR “Emotional Coping” OR “Affect Regulation” OR “Affective Strategy” OR “Psychological Adaptation” OR “Stress Management” OR “Emotional Awareness” OR “Emotional Processing” OR “Adaptive Skills” OR “Emotional Training” OR “Emotion Labelling” OR “Emotional Differentiation” | [18668](https://www-embase-com.ezproxy.lb.polyu.edu.hk/) |
| #4 | 'Emotions'/Exp OR Emotion* OR Psycho* OR Affect* | 375546 |
| #5 | Strateg* OR Tactic* | 113303 |
| #6 | #1 AND #2 AND #3 | 35 |
| #7 | #1 AND #2 AND #4 AND #5 | 23 |
| #8 | #6 OR #7 | 67 |

1. CINAHL Complete (via EbscoHost)

| Search ID# | Search Terms | Results |
| --- | --- | --- |
| S1 | "Neoplasms" OR "Oncology" OR "Tumor" OR "Malignancy" OR "Cancer" | 859,143 |
| S 2 | “Resilience, Psychological” OR “Emotional Resilience” OR “Resilience” | 23,362 |
| S3 | "Emotional Strategy" OR "Emotion Regulation" OR "Emotional Regulation" OR "Emotional Therapy" OR "Mindfulness" OR "Emotion Management" OR "Emotional Coping" OR "Affective Regulation" OR "Affective Strategy" OR "Psychological Adaptation" OR "Stress Management" OR "Emotional Awareness" OR "Emotional Processing" OR "Adaptive Skills" OR "Emotional Training" OR "Emotion Labelling" OR "Emotional Differentiation" | 49,522 |
| S4 | Emotion* OR psycho* OR affect* | 1,364,998 |
| S5 | Strateg* OR tactic* | 386,833 |
| S6 | S1 AND S2 AND S3 | 160 |
| S7 | S1 AND S2 AND S4 AND S5 | 172 |
| S8 | S6 OR S7 | 298 |

1. Scopus

| Search ID# | Search Terms | Results |
| --- | --- | --- |
| #1 | "Neoplasms" OR "Oncology" OR "Tumor" OR "Malignancy" OR "Cancer" | 11,977,617 |
| #2 | “Resilience, Psychological” OR “emotional resilience” OR “resilience” | [954,253](https://www.scopus.com/search/history/results.uri?origin=searchhistory&shid=3) |
| #3 | "emotional strategy" "emotion regulation" OR "emotional regulation" OR "emotional therapy" OR "mindfulness" OR "emotion management" OR "emotional coping" OR "affect regulation" OR "affective strategy" OR "psychological adaptation" OR "stress management" OR "emotional awareness" OR "emotional processing" OR "adaptive skills" OR "Emotional training" OR "Emotion labelling" OR "Emotional differentiation" OR “Emotional” OR “affective” | 657 |
| #4 | Emotion* OR Psycho* OR Affect* | 8,672,199 |
| #5 | Strateg* OR tactic* | 4,718,220 |
| #6 | S1 AND S2 AND S3 | 11 |
| #7 | S1 AND S2 AND S4 AND S5 | 415 |
| #8 | S6 OR S7 | 426 |

1. Web of Science

| Search ID# | Search Terms | Results |
| --- | --- | --- |
| #1 | "Neoplasms" OR "Oncology" OR "Tumor" OR "Malignancy" OR "Cancer" | [5,225,282](https://www.webofscience.com/wos/woscc/summary/e996242a-ed6c-46ea-9112-1f5b7587aea4-011fe5dd3e/relevance/1) |
| #2 | “Resilience, Psychological” OR “Emotional Resilience” OR “Resilience” | [220,424](https://www.webofscience.com/wos/woscc/summary/de46add5-c781-400c-8fc0-40e81aab17cf-011fe5eff9/relevance/1) |
| #3 | "Emotional Strategy" "Emotion Regulation" OR "Emotional Regulation" OR "Emotional Therapy" OR "Mindfulness" OR "Emotion Management" OR "Emotional Coping" OR "Affective Regulation" OR "Affective Strategy" OR "Psychological Adaptation" OR "Stress Management" OR "Emotional Awareness" OR "Emotional Processing" OR "Adaptive Skills" OR "Emotional Training" OR "Emotion Labelling" OR "Emotional Differentiation" | [83,091](https://www.webofscience.com/wos/woscc/summary/d52954f1-26c2-4b70-8c8a-3b4c163eedb8-011fe69499/relevance/1) |
| #4 | Emotion* OR Psycho* OR Affect* | [7,151,398](https://www.webofscience.com/wos/woscc/summary/6a23fcd0-aa3a-4083-ad0c-a1aa97d16e45-011fe6ba0f/relevance/1) |
| #5 | Strateg* OR tactic* | 4,120,143 |
| #6 | S1 AND S2 AND S3 | 250 |
| #7 | S1 AND S2 AND S4 AND S5 | 526 |
| #8 | S6 OR S7 | 721 |

1. CNKI (China National Knowledge Infrastructure)

| Search ID | Search term | Search results |
| --- | --- | --- |
| #1 | (主题：肿瘤) OR (主题：癌症) OR (主题：恶性肿瘤) | 1,591,670 |
| #2 | (主题：心理韧性) OR (主题：心理复原力) OR (主题：复原力) | 6,828 |
| #3 | (主题：情绪调节) OR (主题：情绪管理) OR (主题：情感标签) OR (主题：情感分化) OR (主题：情绪分化) OR (主题：情绪干预) OR (主题：情绪策略) OR (主题：情绪疗法) OR (主题：情感干预) | 28,075 |
| #4 | (主题：情绪) OR (主题：情感) | 910,643 |
| #5 | (主题：方法) OR (主题：干预) OR (主题：策略) OR (主题：措施) | 18,137,415 |
| #6 | #1 AND #2 AND #3 | 8 |
| #7 | #1 AND #2 AND #4 AND #5 | 75 |
| #8 | #6 OR #7 | 77 |

1. Wanfang Database

| Search ID | Search term | Search results |
| --- | --- | --- |
| #1 | 主题:(肿瘤 ) OR 主题:(癌症) OR 主题:(恶性肿瘤) | 3,541,034 |
| #2 | 主题:(心理韧性) OR 主题:(心理复原力) OR 主题:(复原力) | 17,320 |
| #3 | (主题：情绪调节) OR (主题：情绪管理) OR (主题：情感标签) OR (主题：情感分化) OR (主题：情绪分化) OR (主题：情绪干预) OR (主题：情绪策略) OR (主题：情绪疗法) OR (主题：情感干预) | 223,172 |
| #4 | 主题:(情绪) OR 主题:(情感) | 3,002,565 |
| #5 | 主题:(方法) OR 主题:(干预) OR 主题:(策略) OR 主题:(措施) | 29,858,854 |
| #6 | #1 AND #2 AND #3 | 106 |
| #7 | #1 AND #2 AND #4 AND #5 | 148 |
| #8 | #6 OR #7 | 139 |

1. VIPC

| Search ID | Search term | Search results |
| --- | --- | --- |
| #1 | 肿瘤 OR 癌症 OR 恶性肿瘤 | 2,026,543 |
| #2 | 心理韧性 OR 心理复原力 OR 复原力 | 5,956 |
| #3 | 情绪调节 OR 情绪管理 OR 情感应对 OR 情感觉察 OR 情感标签 OR 情感管理 OR 情感分化 OR 情绪分化 OR情感觉察 OR 正念 OR 情绪干预OR 情感策略 OR 情绪策略 OR 情感疗法 OR 情绪治疗 | 91,251 |
| #6 | #1 AND #2 AND #3 | 36 |

1. SinoMed

| Search ID | Search term | Search results |
| --- | --- | --- |
| #1 | "肿瘤"[不加权:扩展] | [1,673,266](javascript:void(0);) |
| #2 | ("情绪"[不加权:扩展]) OR "情感"[不加权:扩展] | [1517](javascript:void(0);) |
| #3 | 情绪调节 OR 情绪管理 OR 情感应对 OR 情感觉察 OR 情感标签 OR 情感管理 OR 情感分化 OR 情绪分化 OR情感觉察 OR 正念 OR 情绪干预OR 情感策略 OR 情绪策略 OR 情感疗法 OR 情绪治疗 | [7250](javascript:void(0);) |
| #4 | ("情绪"[不加权:扩展]) OR "情感"[不加权:扩展] | [163,357](javascript:void(0);) |
| #5 | ("方法"[全部字段:智能] OR "干预"[全部字段:智能] OR "策略"[全部字段:智能] OR "措施"[全部字段:智能]) | [7,704,768](javascript:void(0);) |
| #6 | #1 AND #2 AND #3 | 13 |
| #7 | #1 AND #2 AND #4 AND #5 | 60 |
| #8 | #6 OR #7 | 67 |
