# Supplementary material for: Emotional strategies to enhance resilience in patients with cancer: A scoping review
Source: Asia Pac J Oncol Nurs. 2025 Aug 22;12:100777. doi: 10.1016/j.apjon.2025.100777 (PMC12802108; doi:10.1016/j.apjon.2025.100777)
Supplement: Multimedia component 2 [file mmc2.docx]

**Appendix B**

**Table B.1 Characteristics of included documents**

| No. | Author(s), year | Country | Article type | Study design | Aims | Participants | Setting | Key findings |
| --- | --- | --- | --- | --- | --- | --- | --- | --- |
| 1 | Alarcón et al., 2019 | Spain | Journal article | Quantitative study (cross-sectional survey) | To analyse the psychometric properties of the 10-item version of the Connor-Davidson Resilience Scale (CD-RISC 10©) in breast cancer patients. | Women who had undergone surgery for breast cancer | Hospital | Women with breast cancer who score higher on resilience also score higher on emotional clarity and repair, that is to say, they perceive themselves as better able to understand their feelings and to manage their negative emotions and focus on the positive. |
| 2 | Baghjari et al., 2017 | Iran | Journal article | Quantitative study (cross-sectional survey) | To find out which one of cognitive emotional regulation strategies is a stronger factor in predicting the resiliency in end-of-life stage of patients with cancer. | Women and men with advanced cancer | Hospital | More resiliency signiﬁcantly and more positive strategies, such as acceptance, positive refocusing, refocus on planning, and putting into perspective appeared to be related with more resiliency in cases with advanced cancer. |
| 3 | Baziliansky & Cohen, 2021 | Israel | Journal article | Quantitative study (cross-sectional survey) | First to identify the extent to which cancer survivors use each of the four emotion regulation patterns and to explore the associations among these patterns in a sample of colorectal cancer survivors. Second to examine how background factors and personal resources (resilience and self-compassion) were associated with the four patterns of emotion regulation. Third to assess similarities and differences between the emotion regulation patterns | Colorectal cancer survivors | Cancer center | The participants allocated to Cluster 2 (Cognitive reappraisal) were higher on both personal resilience and self-compassion resources. Cognitive reappraisal is a conscious process that focuses on generating benign or positive interpretations or perspectives on stressful situations as a way of reducing psychological distress. |
| 4 | Cerezo et al., 2014 | Spain | Journal article | Randomised controlled trial | To assess the effectiveness of positive psychology in treatment for breast cancer | Women with breast cancer | Care association | Participants of the experimental group reported improved self-esteem, emotional intelligence-related abilities, resilience, and optimism, as well as positive affectivity, well-being, and happiness. |
| 5 | Chai et al., 2023 | China | Journal article | Randomised controlled trial | To investigate the effect of emotional reassurance strategy combined with processive staged nursing on the psychological resilience and urinary control ability of prostate cancer patients. | Prostate cancer patients who underwent radical prostatectomy | Hospital | Emotional reassurance strategy combined with processive staged nursing for prostate cancer patients can enhance their psychological resilience and urinary control ability, accelerate the recovery process,improve the urinary incontinence,and prevent the complications. |
| 6 | (Cui & Ma, 2024 | China | Journal article | Randomised controlled trial | To explore the effect of psychological intervention based on self-transcendence theory on patients with lung cancer undergoing chemotherapy. | Patients with lung cancer undergoing chemotherapy | Hospital | Psychological intervention based on self-transcendence theory can improve the hope level and resilience of patients with lung cancer chemotherapy, and reduce negative awareness and disease uncertainty |
| 7 | Darabos et al., 2021 | USA | Journal article | Quantitative study (cross-sectional survey) | To examine the potential of coping through emotional processing (EP) and emotional expression (EE) on psychological distress (i.e., depressive symptoms and FCR) and positive aspects of well-being (i.e., PTG and resilience) among young adults with cancer. | Young adults with cancer (18 – 39 years) | Not reported | Use of emotional expression (EE) was significantly associated with lower resilience…Given our finding of coping through emotional expression (EE) being associated with lower resilience it may be that fostering resilience involves understanding emotions more deeply, rather than explicitly expressing emotions. |
| 8 | Finlay-Jones et al., 2023 | USA | Book | Not Available | In chapter 10, to discuss resilience as the ability to adapt to adversity and difficult life circumstances, particularly in the context of surviving military service and surviving cancer. | Cancer survivors | Not reported | Participants described allowing emotions to arise, as well as releasing them as they come up. This also included taking a balanced perspective, instead of getting lost in negative thoughts. |
| 9 | Gao et al., 2022 | China | Journal article | Randomised controlled trial | To explore the effects of Rational Emotive Behavioural Therapy (REBT) on psychological resilience, sleep quality and overall well-being of postoperative liver cancer patients. | Post-operative liver cancer patients | Hospital | REBT helps patients to differentiate between healthy and unhealthy feelings, to think rationally about the relationship between themselves, others and the environment...by actively solving the problems of emotions, behaviour and feelings, patients can be given strong rational beliefs.... It can improve sleep quality, psychological resilience and general well-being. |
| 10 | Ghorbani et al., 2023 | Slovakia | Conference Paper | Quantitative study (cross-sectional survey) | To investigate the predictive contribution of attitude to life and belief system on self-resilience and psychological toughness of cancer patients about the mediating role of emotion regulation. | Cancer patients | Not reported | Using emotional regulation in the context of stress may be effective in maintaining mental health, reducing negative emotions, and even increasing positive emotions through some strategies (positive refocusing, planning refocusing, positive reappraisal, and broad perspective and acceptance), it will result in increased resilience. |
| 11 | Givi et al., 2022 | Iran | Journal article | Quantitative study (cross-sectional survey) | To assess the pain management model based on rumination, as well as positive and negative emotional regulation, with the mediating role of resilience in women with breast cancer. | Women with breast cancer | Hospitals and clinics | Use of positive emotion regulation strategies (acceptance, positive refocusing, planning to refocus, positive reassessment, numerical importance) can have a positive effect on their resilience. A positive view of the situation, along with positive evaluations and self-talk, will arouse positive emotions and control negative feelings. |
| 12 | Guil et al., 2020 | Spain | Journal article | Quantitative study (cross-sectional survey) | To find the specific processes through which the dimensions of Perceived Emotional Intelligence (PEI) (Emotional Attention, Emotional Clarity, and Mood Repair) can act as a risk or protective factor in the development of resilience. | Breast cancer survivors and healthy controls | Cancer center | The direct effects showed that emotional clarity and mood repair increased resilience levels. T emotional attention played a role in vulnerability, decreasing mood repair, and resilience. |
| 13 | Kamışlı & Gökler, 2021 | Turkey | Journal article | Qualitative Study (Semi-structured interviews) | To describe the experiences of patients with metastatic cancer participating in psychodrama group therapy. | Metastatic cancer patients | Hospital | Participants expressed increased psychological resilience, which might be due to the fact that they trusted, accepted, and understood others, received emotional and social support, remembered that they were human, and had unique moments, such as being in a safe haven. |
| 14 | Kang et al., 2017 | China | Journal article | Randomised controlled trial | To explore the impact of psychological support on psychological resilience and emotion regulation in patients with esophageal cancer. | Esophageal cancer patients | Hospital | The patients were able to talk about their emotions and relieve their stress in the process of talking.... Small group activities, patients learn the specific measures of emotional regulation, so that patients can seriously implement the method of emotional regulation.... Psychological support therapy can significantly improve the psychological resilience and emotional regulation ability of oesophageal cancer patients, and can be promoted and used in clinical practice accordingly. |
| 15 | Klainin-Yobas et al., 2023 | Singapore | Journal article | Protocol of a randomized controlled trial | To examine the effectiveness of a digital mindfulness psychoeducation programme (Digital-mindcan programme) on psychological symptoms among cancer survivors. | Cancer survivors | Cancer center | The Digital-mindcan program…Cover issues tailored for cancer survivors including cancer-related stressors (such as permanent surgery scars, physical symptoms, and side effects of medications) thoughts (recurrent thoughts of cancer experiences), emotions and relationships may be (effective to improve resilience for cancer survivors). |
| 16 | Li C. et al., 2018 | China | Journal article | Qualitative Study (Semi-structured interviews) | To explore the process of resilience development in patients undergoing transarterial chemoembolization (TACE) for hepatocellular carcinoma (HCC). | Liver cancer patients treated with TACE | Hospital | In the interviews, patients often used downward and self-comparisons to generate a sense of self-satisfaction...Expanding Positive Emotions...Most patients derive psychological pleasure from shifting away from negative emotions through distraction, positive thinking, etc. |
| 17 | D. Li et al., 2024 | China | Journal article | Qualitative Study (Semi-structured interviews) | To explore the development process of psychological resilience among adult patients with de novo acute leukemia. | Newly diagnosed patients with acute leukemia (AL) | Hospital | Individuals enhance their psychological resilience through emotional regulation and positive coping, driven by various internal and external protective factors. |
| 18 | Lin et al., 2020 | China | Journal article | Randomised controlled trial | To explored the effects of attention and interpretation therapy (AIT) on improving psychological resilience, cancer-related fatigue (CRF), and negative emotions in patients after colon cancer surgery. | Colon cancer patients undergo surgery | Hospital | AIT may improve the psychological resilience of patients after colon cancer surgery in several ways…By learning a variety of new skills (forgiveness, acceptance and self-realization therapy, etc.) …AIT can eliminate negative emotions and stimulate positive emotions, thus relieving psychological pressure. |
| 19 | X. Liu et al., 2023 | China | Journal article | Randomised controlled trial | To evaluate the effects of cognitive behavioral and psychological intervention (CBPI) on social adaptation, psychological resilience, and the level of hope in patients with nasopharyngeal carcinoma (NPC) in radiotherapy | Nasopharyngeal carcinoma patients who received radiotherapy | Hospital | This study demonstrates the positive effects of CBPI on the psychological resilience of patients receiving radiotherapy for NPC. In this regard, CBPI potentially broadens patients’ understanding of their conditions through negative emotion monitoring and health education and motivates family members to keep company with patients, which helps allay negative emotions and boost confidence in treatment. In addition, progressive relaxation training was designed to divert patients’ attention, aid relaxation, dispel negative emotions, and instill confidence and courage into their minds to overcome adversities. |
| 20 | S. Liu et al., 2023 | China | Journal article | Randomised controlled trial | The Managing Cancer and Living Meaningfully (CALM) intervention has been confrmed to improve anxiety and depression in patients, but the role of resilience is still unclear. This study explores this issue. | Breast cancer | Hospital | A positive impact of the CALM intervention can be seen in improved resilience and reduced anxiety and depression, supporting its use as an efective psychological management tool and intervention strategy in the early stages of long-term breast cancer recovery. |
| 21 | Macía et al., 2020 | Spain | Journal article | Quantitative study (cross-sectional survey) | To explore which coping strategies are the most used, in order to know whether different groups of levels of resilience and an appropriate coping style are related to a higher quality of life and better adaptation to the disease. | Cancer survivors | Cancer association | People with higher resilience showed higher scores in the use of adaptive strategies, being acceptance and positive revaluation the most frequent ones |
| 22 | Macía et al., 2021 | Spain | Journal article | Quantitative study (cross-sectional survey) | To explore the underlying association between these aspects for the better understanding of the effect of psychosocial variables on mental health in cancer. | Cancer survivors | Cancer association | The Structural model in the oncological sample showed that adaptive coping was related to resilience, which was directly linked in a negative way to health in people with cancer. |
| 23 | Manne et al., 2015 | USA | Journal article | Quantitative study (cross-sectional survey) | To examine three coping strategies - expressing positive emotions, positive reframing of the cancer experience, and cultivating a sense of peace and meaning in life as potential mechanisms by which resilience translates to quality of life among women recently diagnosed with gynecological cancer. | Women diagnosed with gynecological cancer | Hospital | Resilient women may report higher quality of life during gynecological cancer diagnosis because they are more likely to express positive emotions, reframe the experience positively, and cultivate a sense of peace and meaning in their lives. |
| 24 | Mehrabizadeh et al., 2024 | Iran | Journal article | Qualitative Study (Semi-structured interviews) | To advance intervention strategies to improve health outcomes and behaviors | Women had undergone treatment for breast cancer. | Home and medical center | Among the coping strategies observed in individuals facing breast cancer, both problem-focused and emotion-focused approaches (Emotion-Focused Coping, are employed to manage the illness’s challenges, Emotional Venting and Support from Family Members) are employed to manage the illness’s challenges. |
| 25 | Quan, 2019 | China | Journal | Randomised controlled trial | To explore the effects of a strengthening emotion regulation skills intervention on psychological resilience and coping styles of early gastric cancer (EGC) patients | Early Gastric Cancer | Hospital | Strengthening emotional regulation intervention can effectively improve the negative emotions of early gastric cancer patients, improve the level of social support and psychological resilience of patients, and rationalise patients‘ coping with the disease, which can effectively improve patients’ quality of life and satisfaction with nursing care, and is worthy of clinical promotion. |
| 26 | Walton & Lee, 2023 | India | Journal article | Mixed-methods approach | Phase I: The primary objective of this study was as follows: (1) To identify common protective resilient factors that enabled the adult female cancer survivors to cope with the cancer experience. (2) To identify potential barriers to the resilience of adult female cancer survivors. Secondary objective – Phase II: The secondary objective of this study was to develop and validate a resilience tool for cancer survivorship. | Female breast cancer survivors | Hospital | The categories of themes identified are presented under two main headings, that is, protective resilience factors and barriers to resilience. The theme categories identified under protective resilience factors were personal, social, spiritual, physical, economic and psychological factors. The barriers to resilience identified were lack of awareness, medical/biological barriers, social, financial and psychological barriers. |
| 27 | Wang et al., 2021 | China | Journal article | Randomised controlled trial | To analyze the effect of written expression on self-efficacy, physical symptoms and mental health of young breast cancer patients | Young women with breast cancer (18-30 years) | Hospital | The present study found that 1 month after the intervention, the resilience score of the observation group was higher than that of the control group... The written expression was guided by the patients... Exploring positive emotions, promoting emotional adjustment and adaptation, discovering the positive effects of traumatic experiences, and curbing negative emotions. |
| 28 | Wu et al., 2018 | China | Journal article | Qualitative Study (Semi-structured interviews) | To explore the protective factors of psychological resilience among patients with breast cancer | Breast cancer patients | Hospital | Protective factors of resilience included personal ability (optimistic attitude, firm belief and strong perseverance), disease attribution, social support (information support, behavior and emotional support), social comparison (downward comparison and upward comparison), the meaning of life (hope and helping others) and coping style (confrontation, avoidance, emotional catharsis and fatalistic coping). |
| 29 | Ye et al., 2016 | China | Journal article | Randomised controlled trial | To examined the efficacy of a multidiscipline mentor-based program, Be Resilient to Breast Cancer (BRBC), delivered after breast surgery to (a) increase protective factors of social support, hope for the future, etc.; (b) decrease risk factors of Physical and Emotional Distress; and (c) increase outcomes of Resilience, Transcendence and Quality of Life (QOL). | Breast cancer patients 4 weeks after breast surgery | Hospital | The BRBC intervention improves the positive health outcomes and decreases the risk factors of illness-related distress of breast cancer patients during the high-risk cancer treatment... (intervention will help patients understand their feelings with) Presentation of Anxiety, Depression, Illness Uncertainty, etc. By psychologists |
| 30 | Zhang X. et al., 2024 | China | Journal article | Randomised controlled trial | To investigate the effects of precision nutrition combined with emotional release intervention on nutritional level, immune function and mental toughness of patients underwent laparoscopic radical surgery for colorectal cancer. | Colorectal cancer patients underwent surgery | Hospital | The emotional release part of the intervention can stimulate patients to generate psychological stress through emotional focusing, and then release negative emotions by tapping on the acupoints, activate patients' self-protection consciousness by applying fixed cues, get rid of negative emotions, relax tense nerves, establish positive beliefs, and improve psychological resilience. |
| 31 | Zhang Y. et al., 2023 | China | Journal article | Randomised controlled trial | To explore the effects of psychological intervention based on emotional adaptation theory combined with group cognitive behavioral intervention on fear of disease progression, psychological resilience and complications of breast cancer patients after radical mastectomy. | Patients with breast cancer after surgery | Hospital | Psychological interventions based on affective adaptation theory increase the awareness of breast cancer patients in the early stages of the disease... It enables patients to release their negative emotions, and at the same time enhances their belief in treatment and increases their psychological resilience. |
| 32 | Zhou et al., 2019 | China | Journal article | Randomised controlled trial | To examine whether a cyclic adjustment training (CAT) intervention delivered via a mobile device can improve psychological resilience, and reduce depression and anxiety symptoms, in a population of post-surgical women with breast cance | Women with breast cancer | Hospital | Patients in the CAT group displayed significantly improved psychological resilience, anxiety, and depression scores, respectively, compared to the control group. |
| 33 | Zhu, 2024 | China | Journal article | Randomised controlled trial | To explore the effectiveness of psychological nursing based on the theory of body and mind language programming for ovarian cancer patients, and analyze the impact on their psychological resilience, cancer-related fatigue, and coping strategies. | Ovarian cancer patients | Hospital | Mind-Body Linguistic Programming... Can adjust the patient's negative beliefs and values, and can address behavioural and emotional problems... It can effectively improve the symptoms of cancer-related fatigue and psychological resilience. |

**Table B.2 Quality Assessment/ Appraisal of Analytical Cross-Sectional Studies**

|  | JBI Critical Appraisal Checklist for Analytical Cross Sectional Studies | | | | | | | | |
| --- | --- | --- | --- | --- | --- | --- | --- | --- | --- |
| Author(s), year | 1. Were the criteria for inclusion in the sample clearly defined? | 2. Were the study sub-jects and the setting described in detail? | 3. Was the exposure measured in a valid and reliable way? | 4. Were object-tive, standard criteria used for measurement of the condition? | 5. Were confounding factors identified? | 6. Were strategies to deal with confounding factors stated? | 7. Were the outcomes measured in a valid and reliable way? | 8. Was appropriate statistical analysis used? | Quality score ( number of ‘yes’ choices/total question count) |
| Alarcón et al., 2019 | Yes | Yes | Yes | Yes | Unclear | Unclear | Yes | Yes | 6/8 |
| Baghjari et al., 2017 | Yes | Yes | Yes | Yes | No | No | Yes | Unclear | 5/8 |
| Baziliansky & Cohen, 2021 | Yes | Yes | Yes | Yes | Yes | Yes | Yes | Yes | 8/8 |
| Darabos et al., 2021 | Unclear | Yes | Yes | Yes | Yes | Yes | Yes | Yes | 7/8 |
| Ghorbani et al., 2023 | Unclear | Unclear | Yes | Unclear | Unclear | Unclear | Yes | Yes | 3/8 |
| Givi et al., 2022 | Yes | Yes | Yes | Yes | Unclear | Unclear | Yes | Yes | 6/8 |
| Guil et al., 2020 | Yes | Yes | Yes | Yes | Yes | Yes | Yes | Yes | 8/8 |
| Macía et al., 2020 | Unclear | Yes | Yes | Yes | Yes | Unclear | Yes | Yes | 6/8 |
| Macía et al., 2021 | Yes | Yes | Yes | Yes | No | No | Yes | Yes | 6/8 |
| Manne et al., 2015 | Yes | Yes | Yes | Yes | Yes | Yes | Yes | Yes | 8/8 |

**Table B.3 Supplementary Table 3 Quality Assessment/ Appraisal of Qualitative Research and Mixed Methods Research**

|  | JBI Critical Appraisal Checklist for Qualitative Research | | | | | | | | | | | | | | | | | |
| --- | --- | --- | --- | --- | --- | --- | --- | --- | --- | --- | --- | --- | --- | --- | --- | --- | --- | --- |
| Author(s), year | 1. Is there congruity between the stated philosophical perspective and the research methodology? | 2. Is there congruity between the research methodology and the research question or objectives? | | 3. Is there congruity between the research methodology and the methods used to collect data? | | 4. Is there congruity between the research methodology and the representation and analysis of data? | 5. Is there congruity between the research methodology and the interpretation of results? | | 6. Is there a statement locating the researcher culturally or theoretically? | | 7. Is the influence of the researcher on the research, and vice- versa, addressed? | | 8. Are participants, and their voices, adequately represented? | 9. Is the research ethical according to current criteria or, for recent studies, and is there evidence of ethical approval by an appropriate body? | | 10. Do the conclusions drawn in the research report flow from the analysis, or interpretation, of the data? | | Quality score ( number of ‘yes’ choices/total question count) |
| Kamışlı & Gökler, 2021 | Yes | Yes | | Yes | | Yes | Yes | | Yes | | No | | Yes | Yes | | Yes | | 9/10 |
| Li C. Et al., 2018 | Unclear | Yes | | Yes | | Yes | Yes | | No | | No | | Unclear | No | | Yes | | 5/10 |
| D. Li et al., 2024 | Yes | Yes | | Yes | | Yes | Yes | | Yes | | Yes | | Yes | Yes | | Yes | | 10/10 |
| Mehrabizadeh et al., 2024 | Yes | Yes | | Yes | | Yes | Yes | | Yes | | Yes | | Yes | Yes | | Yes | | 10/10 |
| Wu et al., 2018 | Unclear | Unclear | | No | | Yes | Yes | | No | | No | | Unclear | Unclear | | Yes | | 3/10 |
|  | Mixed Methods Appraisal Tool (MMAT), version 2018 | | | | | | | | | | | | | | | | | |
|  | S1. Are there clear research questions? | | S2. Do the collected data allow to address the research questions? | | 1. Is there an adequate rationale for using a mixed methods design to address the research question? | | | 2. Are the different components of the study effectively integrated to answer the research question? | | 3. Are the outputs of the integration of qualitative and quantitative components adequately interpreted? | | 4. Are divergences and inconsistencies between quantitative and qualitative results adequately addressed? | | | 5. Do the different components of the study adhere to the quality criteria of each tradition of the methods involved? | | Quality score ( number of ‘yes’ choices/ total number of questions specifically evaluating mixed-methods research) | |
| Walton & Lee, 2023 | Yes | | Yes | | Yes | | | Yes | | Yes | | No | | | Yes | | 4/5 | |

**
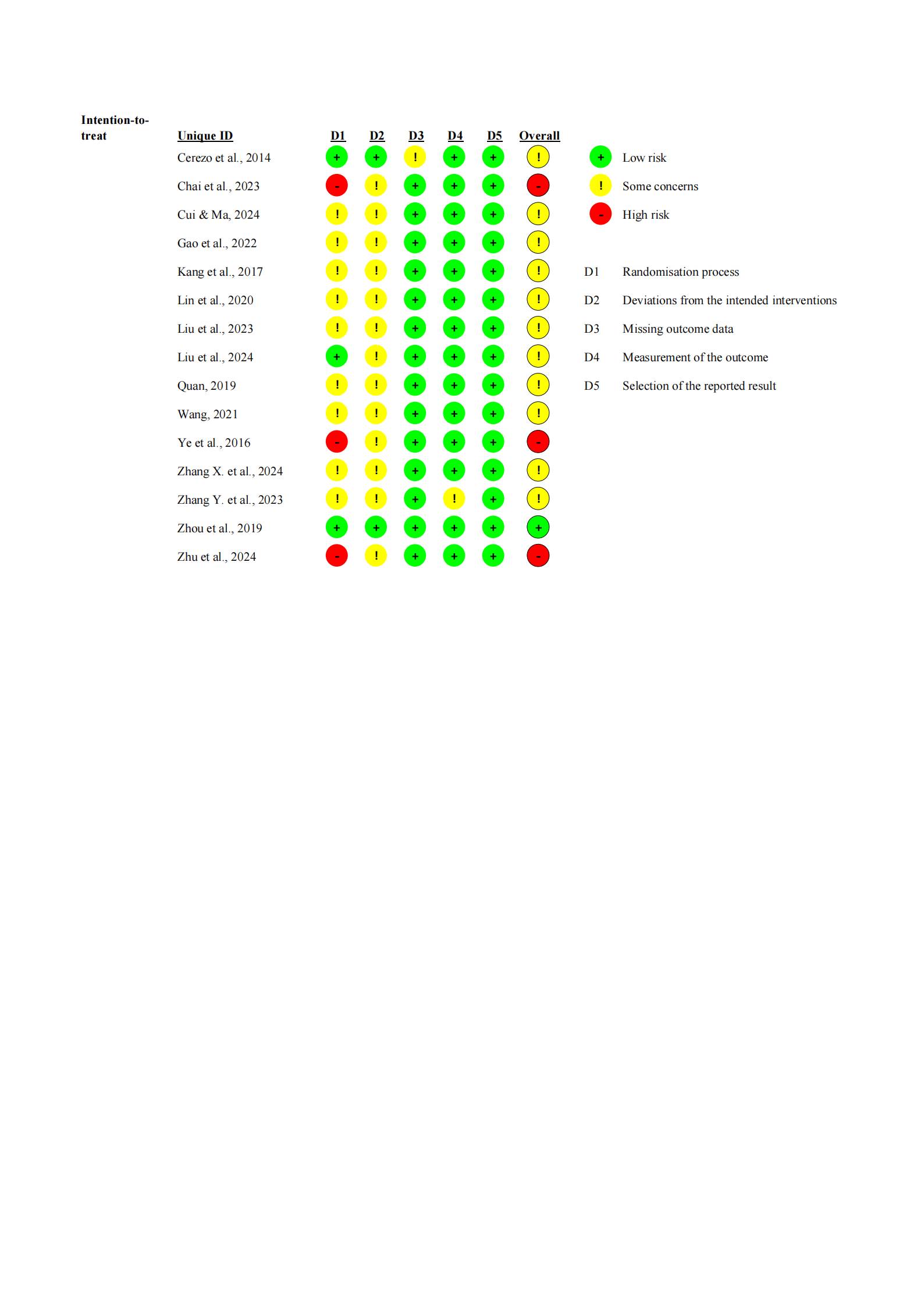
Figure B.1 Cochrane risk of bias in the included randomised controlled studies and protocol of randomised controlled study**


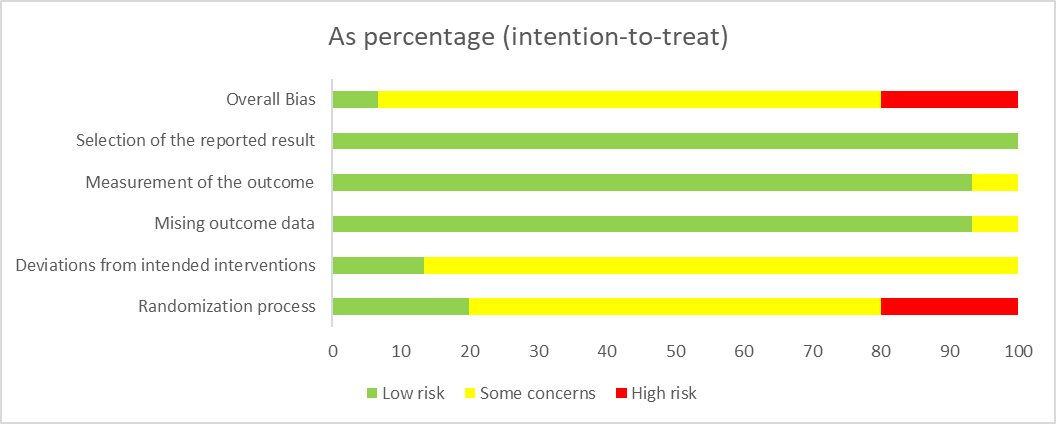


**Figure B.2 Details of each dimension of the Cochrane risk of bias tool used for randomised controlled studies**
