# Supplementary material for: Emotional strategies to enhance resilience in patients with cancer: A scoping review
Source: Asia Pac J Oncol Nurs. 2025 Aug 22;12:100777. doi: 10.1016/j.apjon.2025.100777 (PMC12802108; doi:10.1016/j.apjon.2025.100777)
Supplement: Multimedia component 3 [file mmc3.docx]

**Appendix C**

**Table C Description of the included documents used to do thematic analyses of resilience-enhancing emotional strategies**

| Reference | Emotion identification | | | | Effective emotion regulation | | | | | Emotional support from others |
| --- | --- | --- | --- | --- | --- | --- | --- | --- | --- | --- |
|  | Emotional clarity and repair capacity | Express emotions | Not express emotions | Understand feelings | Positively manage emotions | Cognitive reappraisal | Not self-blame and rumination | Reflection and dynamic adjustment | Release emotions |  |
| Alarcón et al., 2019 |  |  |  | Women with breast cancer who score higher on resilience also score higher on emotional clarity and repair, that is to say, they perceive themselves as better able to understand their feelings and to manage their negative emotions and focus on the positive. |  |  |  |  |  |  |
| Baghjari et al., 2017 |  |  |  |  | More resiliency signiﬁcantly and more positive strategies, such as acceptance, positive refocusing, refocus on planning, and putting into perspective appeared to be related with more resiliency in cases with advanced cancer. |  |  |  |  |  |
| Baziliansky & Cohen, 2021 |  |  |  |  |  | This is supported by the findings that the participants allocated to Cluster 2 (Cognitive reappraisal) were higher on both personal resilience and self-compassion resources. Cognitive reappraisal is a conscious process that focuses on generating benign or positive interpretations or perspectives on stressful situations as a way of reducing psychological distress. |  |  |  |  |
| Cerezo et al., 2014 |  | Self-awareness, and adequate emotional expression to improve emotional and interpersonal communication. |  |  | Promoting change towards positive attitudes, introducing emotional communication skills and assertiveness, adequate expression and repair of negative emotions...Humor enhancement and gratitude techniques were also included. |  |  |  |  |  |
| Chai et al., 2023 |  |  |  | Guiding patients to analyse their emotions: semi-open-ended questioning to guide patients to talk about their inner truths |  |  |  |  |  | Family emotional comfort: Tell family members to not only accompany the patient, but also focus on emotional support and share positive anecdotes.Peer support: Invite patients who have recovered well from radical prostatectomy to give presentations and organise patient exchanges to share their experiences. |
| Cui & Ma, 2024 |  |  |  |  | Self-perpetuating anticipation and forward momentum |  |  |  |  | (Guide the patient to express feelings and gratitude for the people and things) around them. |
| Darabos et al., 2021 |  |  | Further, use of emotional expression (EE) was significantly associated with lower resilience. Given these patterns of findings, it is important to consider that the benefits of EE often do not occur in a vacuum and the social context surrounding EE plays an important role in facilitating adjustment. | Given our finding of coping through emotional expression (EE) being associated with lower resilience it may be that fostering resilience involves understanding emotions more deeply, rather than explicitly expressing emotions. |  |  |  |  |  |  |
| Finlay-Jones et al., 2023 |  |  |  |  |  |  |  |  | Participants described allowing emotions to arise, as well as releasing them as they come up. This also included taking a balanced perspective, instead of getting lost in negative thoughts. |  |
| Gao et al., 2022 |  | Record the day's emotions and behaviours on a daily basis. |  | To make patients perceive their irrational emotions |  |  |  |  |  |  |
| Ghorbani et al., 2023 |  |  |  |  | In the explanation of this finding, it can be argued that using emotional regulation in the context of stress may be effective in maintaining mental health, reducing negative emotions, and even increasing positive emotions through some strategies (positive refocusing, planning refocusing, positive reappraisal, and broad perspective and acceptance), it will result in increased resilience. |  | On the contrary, people with low emotional regulation ability (self-blame, other-blame, rumination, catastrophizing), in the face of life stress and adjustment, will have a weaker adaptation and, as a result, suffer more from depression, despair, and other negative consequences. |  |  |  |
| Givi et al., 2022 |  |  |  |  | Use of positive emotion regulation strategies (acceptance, positive refocusing, planning to refocus, positive reassessment, numerical importance) can have a positive effect on their resilience. A positive view of the situation, along with positive evaluations and self-talk, will arouse positive emotions and control negative feelings. |  | Negative emotion regulation (self-blame, rumination) (is associated with lower resilience). People with cancer put the blame on themselves and thoughts related to the negative event (disease) race through their minds, intensifying the fear of disease. Since their perception transcends reality, they face severe anxiety that impairs patients' resilience to disease. |  |  |  |
| Guil et al., 2020 | The existence of a statistically signiﬁcant positive correlation between breast cancer survivorship with resilience and mood repair pointed out that women who overcome with the disease are more strength by the illness and repair better their moods...Hence, our results reinforce research that demonstrates that women who perceive their emotions clearly and trust in their abilities to repair their emotional states are more strengthened and resilient |  |  |  |  |  |  |  |  |  |
| Kamışlı & Gökler, 2021 |  |  |  |  |  |  |  |  |  | In this study, the participants shared their traumas, which helped other participants analyze and accept their own feelings of death, loss, and mourning. They expressed increased psychological resilience, which might be due to the fact that they trusted, accepted, and understood others, received emotional and social support, remembered that they were human, and had unique moments, such as being in a safe haven. |
| Kang et al., 2017 |  | Encourage patients to vent their emotions and express their deepest feelings. |  |  |  |  |  |  |  | Postoperatively (nurses) encourage patients, stabilise patients' emotions, make patients correctly understand their own disease conditions and the treatment process, patiently listen to patients, enhance patients' confidence, enable family members to give patients appropriate comfort and support, and give specific and effective guidance to patients' concerns, so as to make patients feel at ease and effectively cooperate with the treatment. |
| Klainin-Yobas et al., 2023 |  |  |  |  | Mindful emotions: Calmness and composure...Mindfulness of positive emotions |  |  |  |  |  |
| Li C. et al., 2018 |  |  |  |  | In the interviews, patients often used downward and self-comparisons to generate a sense of self-satisfaction...Expanding Positive Emotions...Most patients derive psychological pleasure from shifting away from negative emotions through distraction, positive thinking, etc. |  |  |  |  |  |
| D. Li et al., 2024 |  | Emotional Regulation: Self-Expression and self adjustment. Self-expression refers to an individual expressing their feelings and emotions in writing or verbally [24]. Self-expression contributes to the development of patients' psychological resilience in terms of emotional release, establishing support systems, information acquisition, self-identification and acceptance, and reflection and meaning construction. |  |  |  |  |  |  |  | The interview responses indicated that patients received social support predominantly in the form of emotional, informational, behavioral, and financial support. |
| (Lin et al., 2020 |  |  |  |  | Introduce ways to vent and regulate emotions, such as listening to music, talking, and writing emotional diaries |  |  |  |  | In this study, the participants shared their traumas, which helped other participants analyze and accept their own feelings of death, loss, and mourning. They expressed increased psychological resilience, which might be due to the fact that they trusted, accepted, and understood others, received emotional and social support, remembered that they were human, and had unique moments, such as being in a safe haven. |
| X. Liu et al., 2023 |  | Psychological counseling was given to patients to understand their emotional needs, encourage the direct expression of negative emotions |  |  | Provide necessary interpretation and guidance according to individual trait emotions. |  |  |  |  | Patient mutual support groups were set up to facilitate communication between patients, further bolster confidence in radiotherapy, and improve compliance with prescribed medication by those who were administered analgesics due to intense pain during radiotherapy |
| S. Liu et al., 2023 |  | The therapist may enhance the capacity of patients and their partners to share their emotional experiences. |  |  | By attending to the emotional state of the patient and demonstrating comfort with emotional distress, the therapist may help the patient to build confidence and a greater capacity to manage emotions. The intent is to support emotional communication and to protect patients from emotional hyperarousal or emotional detachment and numbing. |  |  |  |  |  |
| Macía et al., 2020 |  |  |  |  | The five dimensions of adaptive coping strategies [acceptance, focusing on the planning, positive refocusing, positive revaluation, putting the situation into perspective] showed statistically positive significant differences between resilience groups |  |  |  |  |  |
| Macía et al., 2021 |  |  |  |  | Adaptive coping [acceptance, focusing on the planning, positive refocusing, positive revaluation, putting the situation into perspective] was positively related to resilience |  | Disadaptive coping (self-blame, blaming others=, rumination and catastrophism) was negatively linked to resilience |  |  |  |
| Manne et al., 2015 |  | Resilient women reported using positive emotional expression |  |  |  | Resilient women reported using positive emotional expression, positive reappraisal, |  |  |  |  |
| Mehrabizadeh et al., 2024 |  |  |  |  | Emotion-focused coping often involves minimizing the severity of symptoms and viewing the disease as temporary, even when experiencing significant discomfort. |  |  |  | Emotional venting is used to manage the stress related to social acceptance and appearance changes. Participants expressed concerns about being judged by others due to their altered appearance. | Emotional support from family and healthcare providers is crucial in the recovery process...Family support is a crucial factor in coping with breast cancer, providing emotional comfort and reassurance. However, some participants experienced neglect or a lack of support from family members, highlighting the need for better understanding and assistance. |
| Quan, 2019 |  |  |  |  |  |  |  |  | Emotion therapy: Choose a quiet and comfortable treatment environment, have a professional psychiatrist guide the patient to completely release his/her emotions, soothe the patient's suppressed emotions, make the patient completely relaxed, guide the patient to talk about his/her inner boredom and pressure, correct his/her irrational cognition, beliefs, and viewpoints, and inculcate scientific, healthy, and rational concepts, and regulate his/her bad emotions. | Reconstruction of patients' social support network: health education for patients' families, popularising the psychological characteristics of cancer patients, advising family members to take the initiative to strengthen communication with patients, organising more family activities to reduce the patients' psychological burden; organising regular patient exchanges, encouraging patients to share their experiences of illness, treatment and self-care, eliminating the patients' sense of helplessness and deepening their feelings for each other, with nurses in charge of the patients' lives during the process.In this process, the nurse in charge will affirm and encourage the patients' merits in daily life, strengthen the patients' sense of self-identity, guide the patients to maintain a positive and optimistic attitude towards treatment, and create a sunny and harmonious team atmosphere. |
| Walton & Lee, 2023 |  | (Barriers to resilience) Some participants expressed that they preferred not to talk to close family members for fear of further tension or burden to them. They sometimes felt that family members did not understand what they were going through. |  |  |  |  |  |  | Release of emotions All participants expressed that they did cry at some point of time during the illness. ‘I cried when I got the news and I prayed. I asked God to give me the strength and grace to bear it. I got lots of support and energy’. A few of them spoke about humor. They expressed that laughing and joking helped them to get rid of the negative feelings and created positive energy in them. Avoiding negative thoughts and keeping oneself happy all the time was one of the ways to overcome the disease. |  |
| Wang et al., 2021 |  | Day 1 is for emotional expression, i.e. "Write about the thoughts, feelings and experiences that have touched you the most since you got sick". |  |  |  |  |  | Day 3 Discovering the benefits, i.e. "What positive impacts and changes have you felt since you got sick, what difficulties have you overcome, what new insights have you gained about life". |  |  |
| Wu et al., 2018 |  |  | In order to minimise the impact of cancer on family members, resilient patients generally adopt avoidance strategies, concealing their illness, symptoms and needs from family and friends, especially the elderly and children. |  | Optimism buffers the adverse effects of adversity by enabling individuals to see things in a positive light and to have positive expectations of how things will turn out. |  |  |  | Emotional catharsis: In order to alleviate the effects of their bad moods, some interviewees diverted their attention to other areas by returning to the community, doing household chores, listening to music, watching television, going to the park, chatting, and so on, so as to gradually improve their bad moods. | Emotional support comes from family, medical staff, neighbours, friends, colleagues and patients, with the encouragement of the husband helping the patient to let go of the burden of thought and face the changes brought about by the disease with courage |
| Ye et al., 2016 |  |  |  | (intervention wil help petients understand their feelings with) Presentation of Anxiety, Depression, Illness Uncertainty, etc. By psychologists |  |  |  |  |  | Group discussion among mentors and mentees...Shared trajectory of emotional distress as perceived by mentors and tips to get over them |
| Zhang X. et al., 2024 |  |  |  | Emotional focus, the nurse guides the patient...Recall past painful experiences, such as worries and fears about the disease |  |  |  |  |  |  |
| Zhang Y. et al., 2023 |  |  |  |  |  |  |  |  | Depending on the patient's actual condition and personality, choose the appropriate way to release repressed emotions. | Nursing staff can instruct patients to interact more with other patients...Enhance their health beliefs and treatment confidence. |
| Zhou et al., 2019 |  |  |  | The patient is instructed to self-ask the following questions: Do I have negative feelings right now? If yes, what are they? Do I have positive feelings right now? If yes, what are they? |  |  |  | The patient is instructed to self-ask the following questions: Do I address my negative feelings? If yes, what are they? If no, why? Do I keep and strengthen my positive feelings? If yes, what are they? If no, why? Do I have new negative feelings right now? If yes, what are they? |  | Adjust experiences and feelings sharing with peers |
| Zhu, 2024 |  | Affirm the rationality of patients' negative emotions and encourage them to express the stress caused by work, life and treatment. |  |  |  |  |  |  |  | Family and friend support is equally important. Relatives of patients can be educated and encouraged to participate actively in the patient's treatment, eliminating negative emotions through care and companionship, and helping to create a warm and comfortable environment so as to reduce the patient's stress. |
